# Health Tract, No. 111

**Published:** 1862-10

**Authors:** 


### HEALTH TRACT, No. 111.

### SOLDIER-HEALTH.

The Sanitary Commission have reported that the general rate of disablements by sickness in the army is one hundred and four persons out of one thousand; whereas, only thirteen out of a thousand should be sick at any one time in common life. A Massachusetts regiment, after being a year at the seat of war, has lost no more men from all causes than would have been the case under the ordinary circumstances of home life. A New-York City regiment, after three months of camping, lost but one man out of eight hundred, and he had heart-disease before he left home. But it was a regiment whose average of intelligence and culture was perhaps the highest among the whole Federal force. Both these cases show that camp-life is not necessarily fatal to health or life to a remarkable extent, and that the exercise of an intelligent care on the part of each individual soldier may almost banish disease from an army. And if the officers would coöperate with the men, would encourage them, and do all in their power to facilitate their efforts in this direction, the cost of the war and its duration would be most favorably modified. It is true that only one result is possible, even if Washington were laid in ashes, and the enemy were besieging New-York and Boston—the annihilation of the "Confederacy;" still it is desirable to do this in the shortest time and at the least cost of life and treasure. To bring this about in the most enduring manner, *while the government is* WISELY *waiting on events, until the proper moment arrives for the grand consummating act*, let each soldier for himself, and each soldier's friend at home, and each patriotic officer among too many who are not so! do all that is possible to keep the army in the very highest state of health; because health is efficiency! Just as Lord Nelson's ship was leaving England, he discovered that the flannel shirts of the men were six inches shorter than they ought to have been, and refused to go until the proper kind were furnished. He was ridiculed and called an "old granny." The result was, that while the rest of the fleet was decimated, he did not lose a man! and "his ship, in efficiency, *was as good as* any TWO others"! Aside from the dictates of humanity, a soldier's health should be the highest consideration of any officer who hopes to accomplish great results.

If any one item of health in a soldier is of greater importance than any other, it is a sound condition of the feet. It can not easily be over-estimated. It is the very first of the four great needs of a soldier — good feet, good stomach, good head, and a good heart, that is, loyal to the core, to the authority and honor of his country. To have good feet, there should be good shoes and plenty of walking. A soldier's shoe should not have a high heel, should be made straight on the inside, to give the great toe room to spread out to the fullest; the soles should be abundantly wide, and not too thin. Good food; wearing woolen flannel all over next the skin, winter and summer, the latter most especially; dry bed-clothes; and the most complete ventilation, that is, a constant re-supply of out-door air every second, night and day; these are the grand points to be aimed at, with large fires kindled at sun-down in and around encampments, and kept burning until sunrise. These precautions would save nine out of ten of all the disablements of the army, including both sickness and wounds; for the astounding testimony was given as to the British army in the Crimea, (see SOLDIER-HEALTH, p. 142,) "where one man was under the surgeon's hands for wounds, twelve were under the doctor's for typhus fever, dysentery, and some other diseases brought on by bad food, bad camping arrangements, and the crowding and *avoidable* dissipation of the men."

With these views, we have prepared an abridged edition of 32 pp. of "SOLDIER-HEALTH," (full edition, 25 cts.,) for 5 cts.—40 cts. per dozen, $2.50 per hundred, $20 per thousand—containing upwards of one hundred plain, short, explicit directions for soldiers, given in full in the following pages of the JOURNAL, which those who do not file the numbers can send to friends in the army. Additional copies will be re-furnished to subscribers by mail, at ten cents each.—*Sept.* 2, 1862.

